# Stapled Peptides: An Innovative and Ultimate Future Drug Offering a Highly Powerful and Potent Therapeutic Alternative

**DOI:** 10.3390/biomimetics9090537

**Published:** 2024-09-05

**Authors:** Do-Hee Kim, Sung-Min Kang

**Affiliations:** 1 Research Institute of Pharmaceutical Sciences, College of Pharmacy, Sookmyung Women’s University, Seoul 04310, Republic of Korea; dohee.kim@sookmyung.ac.kr; 2 College of Pharmacy, Duksung Women’s University, Seoul 01369, Republic of Korea

**Keywords:** stapled peptide, protein structure, biomimetics

## Abstract

Peptide-based therapeutics have traditionally faced challenges, including instability in the bloodstream and limited cell membrane permeability. However, recent advancements in α-helix stapled peptide modification techniques have rekindled interest in their efficacy. Notably, these developments ensure a highly effective method for improving peptide stability and enhancing cell membrane penetration. Particularly in the realm of antimicrobial peptides (AMPs), the application of stapled peptide techniques has significantly increased peptide stability and has been successfully applied to many peptides. Furthermore, constraining the secondary structure of peptides has also been proven to enhance their biological activity. In this review, the entire process through which hydrocarbon-stapled antimicrobial peptides attain improved drug-like properties is examined. First, the essential secondary structural elements required for their activity as drugs are validated, specific residues are identified using alanine scanning, and stapling techniques are strategically incorporated at precise locations. Additionally, the mechanisms by which these structure-based stapled peptides function as AMPs are explored, providing a comprehensive and engaging discussion.

## 1. Introduction

The growing resistance of microorganisms to antibiotics is a significant global health concern [1]. Despite the discovery of many new antibiotics, including vancomycin, in the 2000s, humanity continues to suffer from MRSA (Methicillin-Resistant Staphylococcus Aureus) and VRSA (Vancomycin-Resistant Staphylococcus Aureus) infections [2]. The proliferation of drug-resistant strains is largely attributed to the worldwide overuse of antibiotics in humans [3]. Consequently, the current pharmaceutical industry shows great interest in developing new antibiotics targeting these resistant strains [4].

Antibiotics have been heralded as saviors, especially noted for their crucial role during World War II and in combating various diseases [5]. Since the discovery of penicillin in 1928, countless lives have been saved by antibiotics, and they continue to provide new hope to many [6]. However, the emergence of MDR (Multi-Drug-Resistant) strains, including MRSA and VRSA, poses a significant threat to global health care [7]. Additionally, the development of new antibiotics has slowed dramatically since the 20th century, necessitating alternative solutions to combat drug-resistant bacteria [8].

Antimicrobial peptides (AMPs) are relatively short chains, usually comprising 12 to 40 amino acid residues [9]. These peptides typically display a positive charge due to an abundance of basic amino acids such as arginine, lysine, and histidine [10]. Their hydrophobic residues are crucial for penetrating the bacterial membrane [11]. While most α-helical AMPs are unstructured in solution, they adopt a more organized structure upon interacting with bacterial membranes [12]. The evaluation of their preferred secondary structures, antimicrobial activities, and membrane-disrupting capabilities is essential [13]. AMPs can exhibit a micelle-like structure with hydrophobic residues on one side and hydrophilic residues on the other [14]. For example, human antimicrobial peptides such as LL-37 have the ability to form various structures through oligomerization. LL-37 oligomerizes into helical bundles stabilized by hydrophobic interactions and hydrogen bonding between helical segments. Additionally, under certain conditions, it can assemble into fibril-like structures, demonstrating the polymorphism of its oligomerization [15]. Furthermore, the stapling process, as described below, can significantly influence oligomerization and self-assembly. The stapling process increases structural rigidity, facilitating the peptide’s ability to self-assemble into more stable and organized nanostructures. This is closely associated with enhanced antimicrobial activity against pathogens and may play a positive role in amplifying biological activity [16].

The term AMP refers to small polypeptides produced by all living organisms to protect the host from pathogenic microbes, similar to antibiotics [17]. Like antibiotics, AMPs are naturally produced by microorganisms [18]. Due to the vast diversity of microorganisms on Earth, AMPs display remarkable structural and functional diversity and have mechanisms of action different from existing antibiotics [19]. This diversity can make AMPs a valuable alternative to conventional antibiotics for treating MDR bacterial infections [20].

However, AMPs also have undesirable characteristics, such as susceptibility to proteolytic digestion, toxicity to eukaryotic cells, and inefficient delivery to target sites [21]. Solving these issues is crucial for the development of AMPs as new antibiotics [22]. Efforts in the scientific community focus on optimizing AMPs through specific amino acid substitutions, de novo design, and prodrugs to overcome challenges like size reduction and hydrophobicity control [23].

Peptide-based drugs, in general, offer significant advantages, including high bioavailability and flexible conformational structures [24]. Despite their short half-lives due to enzymatic degradation, peptides have potential clinical applications if their proteolytic stability can be improved [25]. Peptides typically have a higher molecular weight (500–5000 Da) than small molecules, offering a larger surface area for interaction with protein targets, leading to fewer side effects and lower toxicity [26]. Additionally, their relatively small size compared to proteins can reduce manufacturing costs [27].

**Figure 1:**
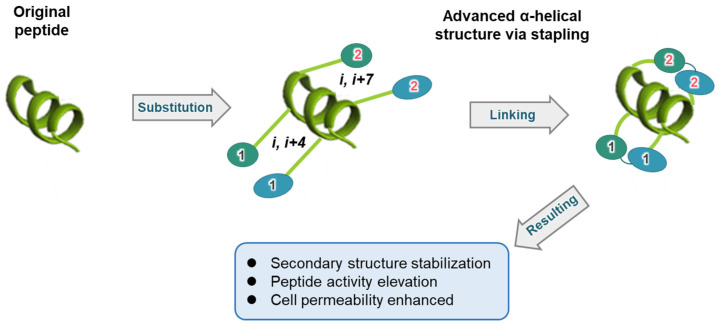
Schematic diagram of peptide helicalization using stapling. The process of the original peptide undergoing helicalization through stapling is depicted. Amino acid residues are denoted by the letter ‘i’, and the types of linkers are indicated using Arabic numerals, with the linked residues connected accordingly.

Various approaches have been proposed to enhance the stability and efficacy of peptide drugs, with peptide stapling being particularly promising [28] (Figure 1). This method involves forming a covalent bridge between amino acid chains, which stabilizes the peptide’s active conformation and protects it from enzymatic degradation [29]. Stapled peptides potentially offer greater drug-like properties than small molecules [30]. Since peptides are usually administered via injection, they can achieve fast systemic absorption, bypass first-pass metabolism, and allow for precise targeting and pharmacokinetic monitoring [31].

Stapled peptides have revolutionized the concept of undruggable targets, overcoming concerns about the large binding interfaces of helical protein fragments compared to small molecules [32]. These techniques enhance the properties of antimicrobial peptides, making them highly efficacious and pathway specific [33]. 

Specifically, hydrocarbon peptide stapling has proven effective in reinforcing α-helicity, improving stability and selectivity [34]. This technique brings previously uncontacted amino acids into appropriate crosslinks, enhancing cell penetration, proteolytic stability, and biological activity [35]. Stapled peptides, particularly in the context of AMPs, stabilize the helical structure and enhance antimicrobial activity [36].

Taken together, AMPs and stapled peptides share a connection through their structural and functional properties. AMPs are short, naturally occurring peptides that play a crucial role in the immune response by disrupting the membranes of pathogenic microorganisms. Similarly, stapled peptides, which are synthetically modified peptides with stabilized α-helices through hydrocarbon staples, enhance proteolytic stability, cell permeability, and target specificity. The structural rigidity and improved bioavailability of stapled peptides make them promising candidates for mimicking the action of AMPs, particularly in targeting membrane proteins and disrupting cellular processes in a similar manner to how AMPs target microbial membranes. Therefore, the design of stapled peptides can be inspired by the functional principles of AMPs, aiming to create potent and selective therapeutics that leverage the inherent antimicrobial mechanisms.

While many reviews discuss stapled peptides, most focus on the mechanical aspects of the stapling strategy. This review aims to minimize redundant explanations and instead emphasize the significance and effectiveness of stapled peptides from a protein structure-based perspective, offering fresh insights into their potential as druggable biomimetics.

## 2. Structure-Based Approach

**Figure 2:**
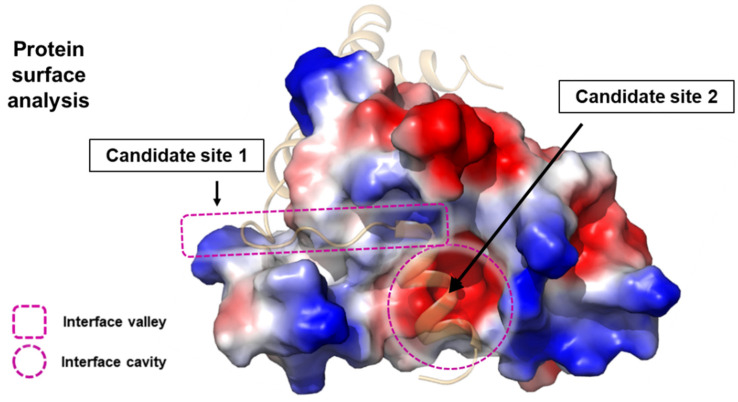
An example of selecting parent peptide positions using protein structure information. Look at the two proteins in the figure, which bind to each other. One protein is depicted in a surface form, while the other is shown as a faint ribbon, revealing its secondary structure. Suppose we need to design a peptide that must bind to the protein displayed in surface form. In that case, Site 1 and Site 2, based on this protein–protein interaction, can serve as effective alternatives by utilizing the interface valley and cavity.

A high-resolution structure can reveal critical positions where introducing a rigid α-helical structure might be essential for tight binding between two proteins (Figure 2) [37]. Such structures provide a detailed view of the molecular interactions and the spatial arrangement of amino acids, allowing for precise identification of regions where structural reinforcement could enhance binding affinity and specificity. If there is an α-helix passing through an obvious pocket or valley within the target protein, this region can be ideal for targeting, as it often represents a key interaction interface [38].

Once the peptide chain that forms the core of the interaction is identified, one can design a stapled peptide consisting of that chain to stabilize its structure and enhance its binding properties [39]. This involves introducing modifications to the peptide, such as incorporating non-natural amino acids or employing specific chemical linkers to create a covalent bond between side chains, thus forming a stable cross-link. These modifications enforce the peptide into an α-helical conformation, which is often more resistant to proteolytic degradation and possesses improved cell permeability compared to its linear counterpart [40].

The process of designing such a stapled peptide typically starts with the selection of suitable sites for modification. Computational modeling and molecular dynamics simulations can be employed to predict the impact of different modifications on the peptide’s structure and function. Once potential sites are identified, synthetic chemistry techniques are used to incorporate the modified amino acids into the peptide chain. The choice of linker type and length is crucial, as it must be compatible with the desired α-helical structure and the specific geometry of the target site [41].

## 3. Selection of Stapling Residues

To stabilize the peptide’s secondary structure, staples must connect two side chains situated on the same face of the helix [37]. It is essential to recognize that the α-helix comprises 3.6 residues per turn. Therefore, the residues selected for stapling should adhere to specific positions: they must be located at i and i+4 (one-loop staple), i+7 (two-loop staple), or even i+11 (three-loop staple) (Figure 1) [28]. When systematically designed in this manner, stapled peptides can enhance protease resistance, improve pharmacokinetic properties, and increase biological activity [42].

As previously mentioned, to induce a peptide to adopt an α-helical structure, it is essential to link the side chains of two amino acids to form a stapled peptide [28]. The number of stapling bridges within the same peptide does not necessarily have to be limited to one. For longer peptides, it is feasible to use double, triple, or even quadruple stapling, utilizing four amino acids to create two distinct side braces. Typically, double stapling alone is sufficient to confer the desired helicity to the peptide, generally achieving over 80% helicity [43].

For the design of hydrocarbon peptide stapling, it is crucial to select residues that are vital for maintaining the helical structure and are located in regions likely to form contact faces (usually lysine) [30]. Since stapling can alter the physicochemical properties of the original amino acids, residues essential for preserving the helical structure are typically excluded from stapling [32]. Generally, the spacing between residues selected for stapling corresponds to one helix turn or two helix turns, which means choosing the i-th residue, the i+4-th residue, and the i+7-th residue. For maximizing helicity, the i+11-th residue may also be chosen. A reasonable approach for determining the residues for stapling involves performing an alanine scanning and using the results to guide the selection process [43].

## 4. Alanine Scanning

An α-helix typically contains anywhere from a few to several dozen amino acids. In a conventional α-helix, each amino acid residue is arranged at an approximate 100° relative to the axis, creating a spiral shape. A single turn of the helix generally includes about 3.6 amino acid residues on average [44].

The region of the α-helix where stapling will be applied is determined through structural analysis, as described in the previous section. To ensure that the implemented peptide functions effectively as a helix when synthesized, it is crucial to select appropriate residues for stapling [45].

For the peptide to interact effectively with its partner, as it does in its native form, it is essential to preserve amino acid residues that are critical for this interaction. In other words, residues significant for recognition should not be modified by the stapling technique [46]. Although each amino acid contributes differently to the formation of the α-helix, methionine, alanine, leucine, glutamate, and lysine are generally vital for helix formation. Thus, the residues chosen for the stapling technique should be those that are essential for maintaining the α-helix structure but are not critical for the interaction [47].

**Figure 3:**
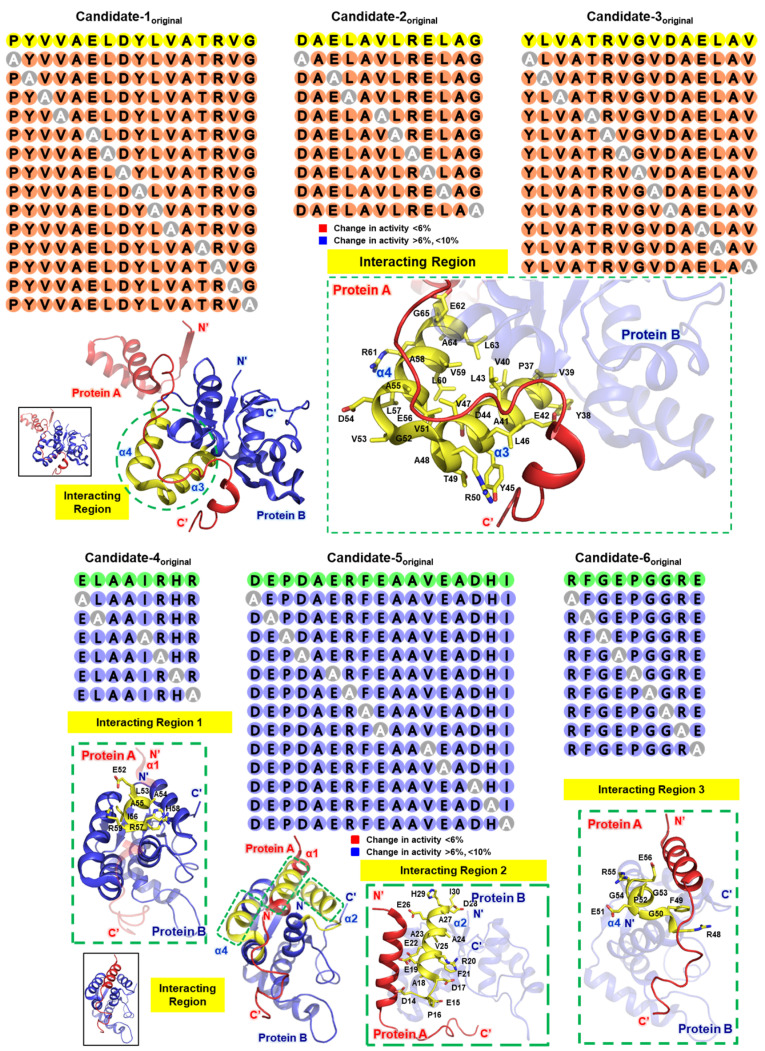
Example of structure-based alanine scanning. For convenience, the proteins used in this description are labeled as A and B. As described in the main text, this process involves identifying stapling spots that maintain the α-helix structure while minimally affecting key interactions. Candidate peptides, in which amino acid residues are sequentially substituted with alanine, are ultimately selected based on the preservation of their activity compared to the parent peptide. This figure was created using the template structures 5X3T and 4XGQ from the Protein Data Bank [48].

Due to the potential discrepancy between theoretical predictions and actual results, it is often beneficial to conduct alanine scanning for thorough validation (Figure 3). Specifically, this involves sequentially replacing each amino acid in the peptide sequence with alanine and assessing the activity of each resulting peptide, using appropriate in vitro or in vivo methods [43]. This approach helps identify the positions where amino acid substitutions have minimal impact on activity, allowing those positions to be designated for the stapling technique [40].

Our argument for the potential of stapled peptides is critically supported by examples based on the VapBC26 and VapBC30 proteins derived from *Mycobacterium tuberculosis*. In this study, researchers designed peptidomimetics to inhibit the VapBC26 and VapBC30 complexes by mimicking their binding interfaces. These peptidomimetics, particularly those mimicking the α3 and α4 helices of VapC26 and the α1 helix of VapB30, successfully disrupted the protein complexes, thereby increasing RNase activity and inhibiting bacterial growth, ultimately enhancing druggability. The peptidomimetics, named ‘V26-SP-8’ and ‘V30-SP-8’, created through α-helix stapling, demonstrated improved cell penetration, stability, and efficacy, even surpassing traditional antibiotics like vancomycin. These findings suggest that stapled peptides could be a promising strategy for developing novel drugs with high druggability [49]. Like this, stapling has proven to be a valuable tool in peptidomimetics by stabilizing the α-helical structure, enhancing proteolytic stability, and improving cell permeability. These modifications increase the therapeutic potential of peptidomimetics, making them more effective AMPs and offering new possibilities in drug design.

## 5. CD Spectroscopy

After selecting residues for stapling through alanine scanning to preserve biological or chemical activity while maintaining α-helicity, it is essential to confirm the formation of the α-helical secondary structure. If amino acids like proline, which disrupt α-helicity, do not significantly impact activity and are located at the ends of the peptide sequence, they may be removed for better results in α-helicity measurements using circular dichroism (CD) spectroscopy [50].

**Figure 4:**
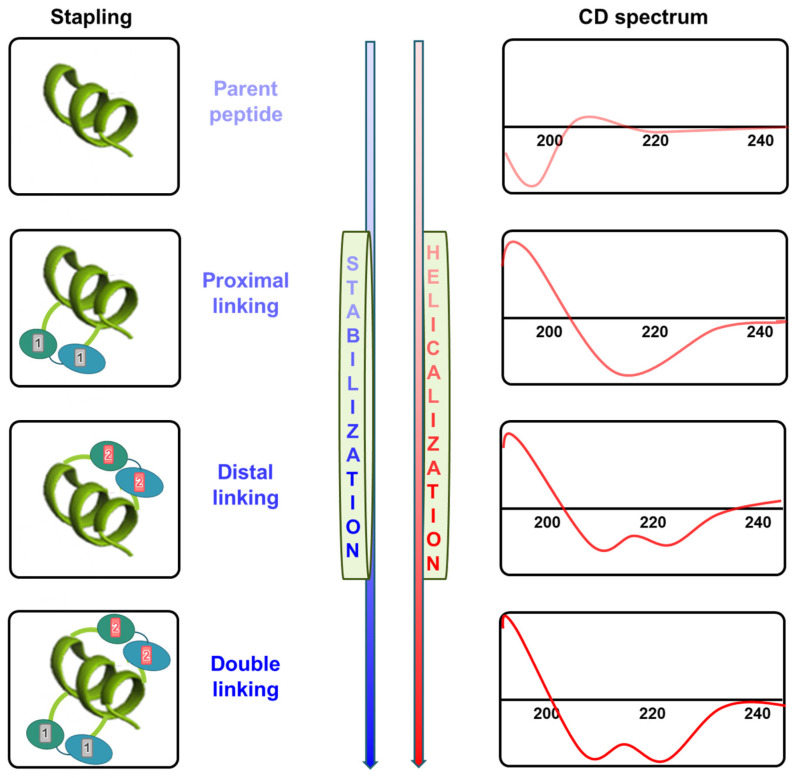
Diagram of stabilization and improved helicity through stapling. The diagram illustrates the stabilization and enhancement of helicity due to stapling. As stabilization progresses, the CD spectrum exhibits two distinct minima. This trend highlights the characteristic improvement of secondary structure clarity with repeated linking.

CD spectroscopy is primarily used to investigate structural changes in stapled peptides and estimate their helicity (Figure 4). Peptides and proteins exhibit distinct CD spectra based on their predominant secondary structure [51]. Peptides in an unstructured conformation show a strong minimum at 195 nm in the CD spectrum, while those in an α-helical conformation typically exhibit a strong positive peak at 190–195 nm and dual minima at 208 and 222 nm. Specifically, the peak at 222 nm is a hallmark feature of the α-helix structure. Therefore, when interpreting CD data, the greater the intensity of the positive peak at 190–195 nm and the negative peaks at 208 nm and 222 nm, the higher the quantitative degree of α-helix formation. For instance, after measuring the CD spectrum, the α-helical content can be quantified by comparing it with standard α-helix reference data, allowing for the estimation of the proportion of α-helical structure present. Successful stapling, especially when more distal regions are linked or multiple sites are stapled, often leads to significant increases in helicity due to α-helix stabilization [52]. This α-helicity can be quantified using simple software like CDNN [53].

## 6. Stability Confirmation Post-Synthesis

To assess the proteolytic stability of stapled peptides, trypsin degradation tests are commonly employed [45]. Trypsin is frequently used in these protease tests because it primarily cleaves at the carboxyl side of charged amino acids such as Lys and Arg [54]. If the results indicate that the stapled peptide exhibits greater stability to protease degradation than its native linear counterparts, the stapling is considered successful [55].

## 7. Conclusions

In drug discovery, proteins that engage in intracellular interactions with other proteins are widely regarded as highly biologically appealing targets. As the building blocks of life, proteins play a pivotal role in all aspects of cellular systems, regulating numerous physiological enzymatic activities through structural stabilization [56]. This is true not only for large proteins but also for small peptides with fewer than 50 amino acids, which perform critical enzymatic activities as antibacterial agents, hormones, and neurotransmitters essential to every living organism [57].

Stapling peptides combine the broad target recognition capabilities of protein therapeutics with robust cell-penetrating ability. The successful design and evaluation of potent stapled peptide interactions demonstrate that stapling can significantly enhance the pharmacologic performance of peptides [30]. This includes increasing target affinity, proteolytic resistance, and serum half-life, while also conferring high levels of cell penetration [45].

Since intracellular protein–protein interaction-derived stapled peptides represent a recent advancement, the successful implementation of stapled peptide technology requires meticulous observation of protein–protein interactions and the optimization of a multistep process. This involves varying the positions and number of staples to determine the optimal output [58].

Stapled peptides offer a novel therapeutic alternative capable of inhibiting the function of proteins, such as enzymes, that were previously difficult to target using classical small molecules. Theoretically, the emergence of an α-helical structure through stapling increases protease resistance by blocking the protease enzyme’s access to target sites on peptide chains, thereby enhancing peptide stability [59]. This ultimately leads to improved delivery success rates for peptide drugs.

Although practical examples are still limited, the growing number of reports on stapled peptides as potent and specific inhibitors of protein–protein interactions suggests that they could provide crucial information for drug development in the future.

## Data Availability

Data are contained within the article.
